# High lipoprotein(a) results in overestimation of BQ-based low-density lipoprotein-cholesterol measurement

**DOI:** 10.1016/j.jlr.2026.101080

**Published:** 2026-06-13

**Authors:** Azusa Yamazaki, Yuna Hakii, Masumi Ai, Shuji Miyake, Junichiro Takahashi, Akira Yoshimoto, Takahiro Kameda, Naoya Ichimura, Shuji Tohda, Shinji Yokoyama, Ryunosuke Ohkawa

**Affiliations:** 1 Clinical Bioanalysis and Molecular Biology, Graduate School of Medical and Dental Sciences, Institute of Science Tokyo, Tokyo, Japan; 2 Clinical Laboratory, Institute of Science Tokyo Hospital, Tokyo, Japan; 3 Insured Medical Care Management, Graduate School of Medical and Dental Sciences, Institute of Science Tokyo, Tokyo, Japan; 4 Health Administration Center of Institute of Science Tokyo, Tokyo, Japan; 5 Immuno-Biological Laboratories Co., Ltd, Gunma, Japan; 6 Clinical Laboratory Science, Faculty of Medical Technology, Teikyo University, Tokyo, Japan; 7 Food and Nutritional Sciences, Chubu University, Kasugai, Japan

**Keywords:** lipoprotein (a), LDL, VLDL, cholesterol, lipoproteins, beta-quantification method, ultracentrifugation, gel permeation high performance liquid chromatography

## Abstract

Most conventional low-density lipoprotein cholesterol (LDL-C) assays are standardized by the CDC reference procedure of beta-quantification (BQ) method, which involves density-based ultracentrifugation to separate very low-density lipoprotein (VLDL) and LDL. Apolipoprotein (apo) (a) covalently binds to apoB, altering lipoprotein density thereby affecting this separation. We evaluated the impact of lipoprotein(a) [Lp(a)] on LDL-C assay accuracy. The distribution of apo(a) between VLDL and LDL was examined by ultracentrifugation and gel permeation high-performance liquid chromatography (GP-HPLC) analysis in three subjects. LDL-C values were compared between the conventional assays calculated by Sampson’s formula (S-LDL-C) and direct homogeneous assay (D-LDL-C) and the GP-HPLC analysis (GP-LDL-C) for 1,560 human samples, and the influence of Lp(a) concentration was analyzed. Apo(a) was detected in the LDL fraction by ultracentrifugation and in both VLDL and LDL fractions by HPLC. Strong correlations were observed between S-LDL-C, D-LDL-C, and GP-LDL-C, but significant differences existed among the methods (*P* < 0.001). The differences between S-LDL-C or D-LDL-C and GP-LDL-C positively correlated with Lp(a) (r = 0.302 and 0.321, respectively, *P* < 0.001). When Lp(a) exceeded 30 mg/dl, these differences were significantly larger (n = 139). The difference between S-LDL-C and D-LDL-C was not significantly correlated with Lp(a) (*P* = 0.774). Conversely, VLDL-C measured by Sampson’s method showed a negative correlation with Lp(a) (r = −0.262, *P* < 0.001) Apo(a) increases VLDL density, causing it to be recovered in the LDL fraction during density-based separation. Consequently, Lp(a) causes concentration-dependent overestimation of BQ-based LDL-C, and underestimation of VLDL-C.

Low-density lipoprotein (LDL)-cholesterol (LDL-C) in serum/plasma is one of the major risk factors for atherosclerotic vascular diseases. It is therefore important to measure its value accurately. LDL is one of the serum lipid-protein particles (lipoproteins) that are originally defined by the density in ultracentrifugation; very low-density lipoprotein (VLDL), LDL and high-density lipoprotein (HDL) as below 1.006 g/ml, between 1.006 and 1.063 g/ml, and between 1.063 and 1.210 g/ml, respectively. VLDL and LDL both contain apolipoprotein (apo) B, but LDL is unique that it contains apoB as its sole protein component. This property allows for the selective sedimentation of apoB-containing lipoproteins with polysaccharides sulfate and divalent cation complex. Using these principles, LDL-C can be measured as follows. Fasting human serum is ultracentrifuged at a density of 1.006 g/ml, and the supernatant is removed as VLDL fraction. The cholesterol content of the bottom fraction that contains both LDL and HDL is measured chemically using the Abell Kendall method (1). The bottom fraction is then subjected to sedimentation of LDL with heparin and manganese chloride, and cholesterol in the supernatant is measured as HDL. LDL-C is calculated as subtraction of HDL-C from cholesterol in the ultracentrifuge bottom fraction. This is now authorized by the CDC as the reference method for LDL-C measurement, established as beta-quantification (BQ) (2, 3). Various methods and formulas to measure serum LDL-C have been developed and standardized against this BQ reference method (4), except for gel-permeation high-performance liquid chromatography (GP-HPLC) (5) and NMR (6).

The equation proposed by Friedewald has been widely used to calculate serum LDL-C from total cholesterol, triglyceride and HDL-C (7). Modification of this equation has been proposed by various authors, most recently by Sampson *et al.* (8, 9). We validated this equation by using GP-HPLC on 496 human samples (10). Each lipoprotein fraction was demonstrated to be highly correlated between the equation and HPLC. However, there is a slight underestimation in VLDL and a slight overestimation in LDL in the calculated values over the HPLC-measured data, while the HDL data match between the direct method and HPLC well (10). Some of the apoB protein is covalently attached by apo(a), which has various molecular weights ranging from 300 to 900 kDa. The lipoprotein particles containing apoB-apo(a) complex are called lipoprotein(a) [Lp(a)] (11). Lp(a) is recognized as an independent strong risk for atherosclerosis. Lp(a) is mostly overlapped with LDL with respect to density classification. However, the large molecule size of apo(a) (300–900 kDa) may affect the molecular weight, particle size and density of the lipoproteins it attaches. We therefore investigated how the presence of apo(a) influences the density and size of lipoproteins, and accordingly, conventional LDL assays validated by the BQ method.

## Materials and Methods

### Simulation of change in density and size of lipoproteins by apo(a) attachment

Potential increase of VLDL density and LDL diameter by attaching apo(a) was calculated based on the chemical composition of VLDL subfractions and LDL according to the simulation by Shen and Kézdy (12). Calculation for change of their density by apo(a) was attempted assuming average molecular weights of amino acid 120, phospholipid 780, cholesterol 387, cholesteryl ester 660, and triglyceride (TG) 880 Da, and average densities of amino acid as 1.34 g/ml, triglyceride as 0.9 g/ml, and other lipid as 1 g/ml, for various molecular weights of apo(a). Increase of LDL Stokes diameter by apo(a) was also estimated similarly.

### Experimental studies

#### Sample collection

Blood samples were collected from three healthy volunteers (Subjects A–C) after fasting for 12 h. The collected blood was left to stand for 15 min to allow coagulation. Serum samples were obtained by centrifugation at 2,000 × *g* for 15 min, and stored at −80°C until use. The following lipid parameters were measured within one year of collection, and samples were subjected to only one freeze-thaw cycle prior to analysis.

#### Laboratory measurements

Total cholesterol (TC) and TG were quantified using commercial enzymatic assay kits: Cholestest CHO and Cholestest TG (SEKISUI MEDICAL, Tokyo, Japan), respectively. HDL-C and LDL-C were determined by “homogeneous phase” assays using Cholestest N HDL and Cholestest LDL (SEKISUI MEDICAL, Tokyo, Japan), respectively. Lp(a) concentration was measured using an assay kit, Lp(a) Latex “DAIICHI”, immunoturbidimetric assay (SEKISUI MEDICAL), using monoclonal antibody against an unknown epitope of apo(a). The data are presented as lipoprotein mass concentration (mg/ml) using a converting factor 4.2 for apo(a) protein to Lp(a) assuming “standard” Lp(a) (13). Since the system uses a monoclonal antibody against apo(a) (of the unknown epitope), the assay values should reflect molecular concentration of apo(a), although the kit displays mass concentration. To further characterize the serum samples from the three healthy volunteers (Subjects A - C), Lp(a) levels and apo(a) isoform sizes were analyzed by Medpace Reference Laboratories. Lp(a) was determined using the Roche Lp(a) Gen2 kit on a Roche c502 analyzer, utilizing molar calibrator sets traceable to the WHO PRM 2B for molar concentration, and the Roche internal standard for mass concentration. Apo(a) isoform size (number of kringle IV [KIV] repeats) and relative expression ratios were determined using a validated electrophoresis-based assay.

#### Separation of lipoproteins by modified BQ method

The sera were fractionated according to BQ method with partial modification. Three milliliters of serum was centrifuged at 542,715 × *g* and 4°C for 20 h with a density of 1.006 g/ml using a TLA-110 rotor (Beckman Coulter). The 1 ml aliquot of the upper layer was collected the top fraction (d < 1.006 g/ml) as the VLDL fraction. The remaining 2 ml bottom layer (d > 1.006 g/ml) was diluted with 1 ml saline. For 200 μl aliquot of this fraction, apoB-containing lipoprotein was precipitated by adding 8 μl of heparin (5,000 USP units/ml heparin sodium; FUJIFILM Wako Pure Chemical Industries) in saline and 10 μl of 1 M manganese(II) chloride (MnCl_2_) solution. After incubation for 30 min at 4°C, the precipitate was removed by centrifugation at 1,500 ×*g* for 30 min at 4°C to obtain HDL fraction as the supernatant. The precipitate was washed twice with saline containing Heparin/MnCl_2_ and finally dissolved in saline by vigorously vortexing. The cholesterol level in the separated VLDL fraction, bottom layer (containing LDL and HDL), and HDL fraction was determined using the commercial enzymatic assay kit Cholestest CHO (SEKISUI MEDICAL). The cholesterol content of the VLDL fraction was defined as BQ-VLDL-C. BQ-LDL-C was calculated by subtracting the HDL-C value from the cholesterol content of the bottom layer.

#### Separation of lipoproteins by GP-HPLC

GP-HPLC analysis was performed using the LipoSEARCH® method (Immuno-Biological Laboratories Co) as a reference with some modifications (5, 10). LipoSEARCH® system was constituted of a Shimadzu Prominence HPLC system (Shimadzu Inc., Japan) equipped with four pumps LC-20AD, an autosampler SIL-20AC, two degassing units DGU-20A, two ultraviolet detectors SPD-20A, and a CBM-20A system controller. Four microliters of filtered serum were injected into the serially connected SkylightPakLP1-AA gel permeation columns (Immuno-Biological Laboratories Co., Ltd., 300 mm × 4.6 mm I.D.) maintained at 25°C and eluted with running buffer at a flow rate of 0.24 ml/min. After separation by particle size, the flow path was split equally into two lines, and cholesterol and TG reagents were merged in each channel at a flow rate of 0.12 ml/min to cause the enzymatic reactions at 37°C. The absorbance at 550 nm was continuously monitored using a dual-detection system to obtain cholesterol and TG chromatograms simultaneously. Cholesterol and TG values of each lipoprotein subclass were calculated by measuring the calibration serum and fitting a Gaussian curve to the chromatograms; chylomicron (>80 nm, 1 and 2), VLDL (30–80 nm, 3 to 7), LDL (16–30 nm, 8 to 13), and HDL (8–16 nm, 14 to 20) (14, 15). The total amounts of cholesterol in each subfraction of VLDL, LDL and HDL were defined as GP-VLDL-C, GP-LDL-C, and GP-HDL-C, respectively. The intra-assay repeatability (n = 20) for this method showed a coefficient of variation of less than 3% (supplemental Table S1). For lipoprotein subclass collection, we injected 8 μl of filtered serum into the GP-HPLC with using running buffer instead of reagents at a flow rate of 0.12 ml/min. Lipoprotein fractionation was performed referencing the method reported previously (16).

#### Analysis of apolipoproteins by electrophoresis and Western blotting (WB)

Sodium dodecyl sulfate polyacrylamide gel electrophoresis (SDS-PAGE) was performed using 4% polyacrylamide running gels and 3% stacking gels under reducing conditions with 100 mM dithiothreitol. The separated proteins were transferred to PVDF membranes (Merck Millipore). After blocking the membrane with 5% skim milk, apo(a) was primarily detected with a monoclonal antibody against an unidentified epitope of apo(a), kindly provided by Sekisui Medical Co. (which is used in the Lp(a) assay kit produced by the company). Horseradish peroxidase (HRP)-conjugated goat anti-mouse IgG antibody (Medical & Biological Laboratories) was used as the secondary antibody. The apo(a) band was visualized using ECL Prime Western Blotting Detection Reagents (Cytiva). After stripping the membrane of the antibodies used to detect apo(a), the apoB band was detected using an anti-apoB polyclonal antibody (Academy Bio-Medical Company) as the primary antibody and an HRP-conjugated rabbit anti-goat IgG antibody (Medical & Biological Laboratories) as the secondary antibody, followed by the ECL detection. For the detection of apoE and apoA-I, 12.5% running gels and 4.5% stacking gels were prepared. Using the same procedure as described above, the primary antibodies were polyclonal antibodies to apoE and apoA-I (both from Academy Bio-Medical Company), and the secondary antibody was the same as that to apoB. ApoA-I was detected by stripping after apoE.

### Bioinformatic analysis

#### Samples and lipid/lipoprotein data

Anonymized test results were obtained from the regular health examination of the employees at Tokyo Medical and Dental University between September 25, 2008, and October 31, 2010 (n = 1,629). After collecting the samples, these were stored immediately at −80°C until used. The following lipid parameters were measured within one year of collection, and samples were subjected to only one freeze-thaw cycle prior to analysis. TC, TG, HDL-C, LDL-C, and Lp(a) were determined using the commercial kits described above (see Laboratory measurements). The LDL-C measured by the above homogeneous direct assay was referred to as D-LDL-C. Based on the values of TC, HDL-C, and TG, LDL-C was calculated using two formula-based methods: Friedewald’s and Sampson’s equations, referred to as F-LDL-C and S-LDL-C, respectively (7, 8). Additionally, VLDL-C level was calculated using Sampson’s equations as S-VLDL-C (17). Note that LDL-C by a homogeneous method, and the calculations are all validated by the BQ method. After excluding 69 participants with missing data or/and of elevated TG levels (≥800 mg/dl), the medical examination data of 1,560 subjects (male: n = 571, age 46.1 ± 9.8; female: n = 989, age 38.0 ± 12.2) were retrospectively analyzed.

### Statistical analyses

All the data were statistically analyzed using SPSS version 25.0 (IBM Corp). The Kolmogorov-Smirnov test was performed to assess whether the data were from a Gaussian distribution, and nonparametric tests were used to evaluate the non-normal data. Correlation analyses were performed using Spearman’s rank correlation test (Figs. 2 and 3). Friedman’s test with Bonferroni correction as a post hoc test was used for the comparison between methods for LDL-C (Fig. 2). Quantitative data on subject baseline characteristics and differences in each LDL-C or VLDL-C between low and high Lp(a) groups were evaluated using Mann-Whitney *U* test (Table 4 and Fig. 3). Qualitative variables were compared using Chi-square and Fisher's exact tests (Table 4). All tests were two-sided, and statistical significance was set at *P* < 0.05.

**Fig. 1:**
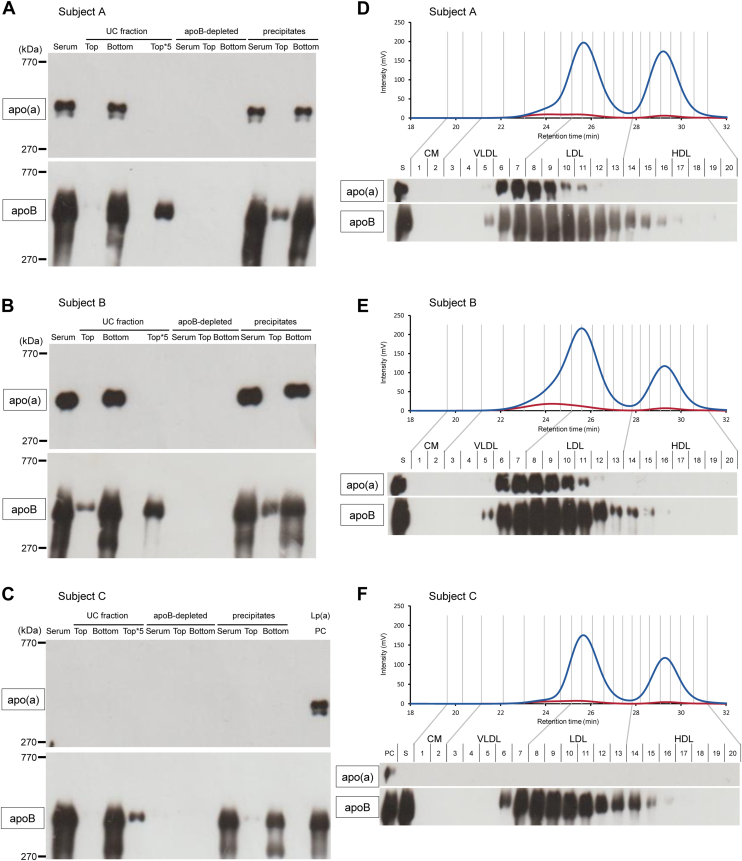
Distribution of apo(a) and apoB in ultracentrifugation and GP-HPLC fractions. Serum was separated into top (d < 1.006 g/ml) and bottom (d > 1.006 g/ml) fractions by ultracentrifugation (UC). The whole serum and both UC-separated fractions (Top and Bottom) were further subjected to heparin-manganese precipitation to obtain apoB-depleted supernatants (labeled "apoB-depleted" in panels A–C) and apoB precipitates (labeled "precipitates" in panels A–C). Each fraction applied at 0.5 μl equivalent to serum was analyzed by SDS-PAGE and WB using anti-apo(a) and apoB antibodies (A–C). Top∗5 is the top fraction concentrated by 5 times. For GP-HPLC, 8 μl of serum was separated into 20 detailed lipoprotein fractions according to previously reported method (16). Each sample (20 μl) from 20 collected fractions were analyzed by SDS-PAGE and WB using anti-apo(a) and apoB antibodies (D–F). Fractions in HPLC corresponding electrophoresis is indicated by lines. Lane "S" contains 0.1 μl of serum as a control. PC in subject C is a control human serum (0.05 μl) with detectable apo(a). The blue and red lines represent the chromatograms of total cholesterol and triglyceride, respectively.

**Fig. 2:**
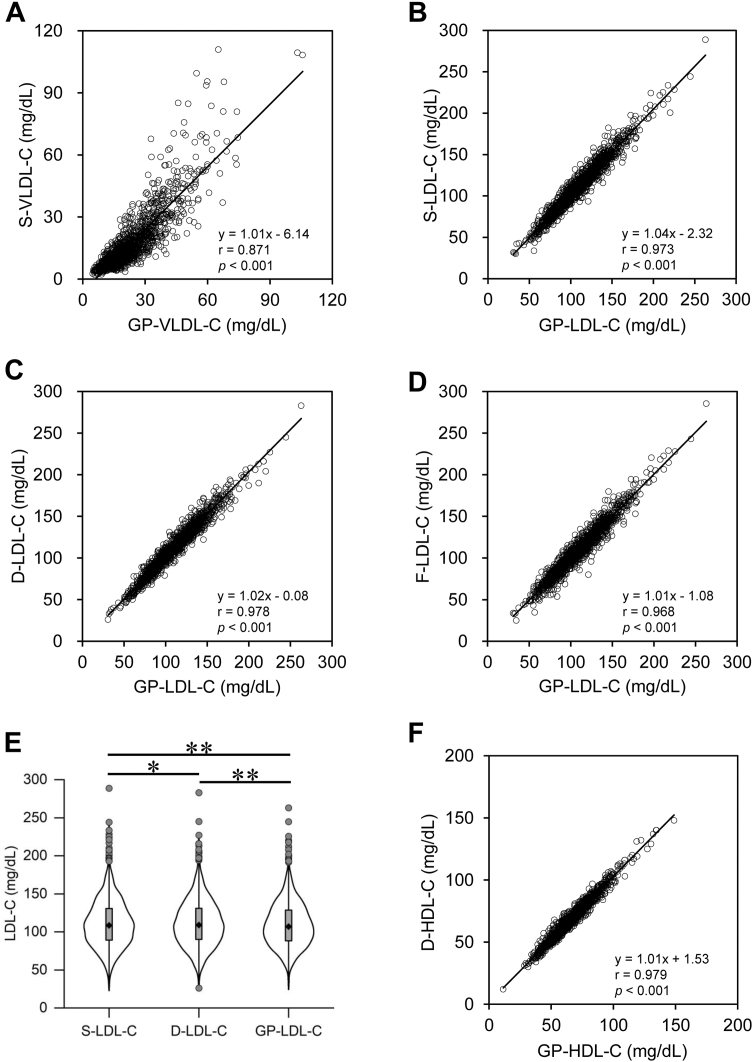
Correlation of lipoprotein cholesterol between HPLC and BQ-based assay. Lipoprotein cholesterol (VLDL-C (A), LDL-C (B–D) and HDL-C (F)) concentrations were compared between the different methods. Correlations were evaluated using Spearman’s rank correlation test. E: The values of LDL-C were compared. ∗*P* < 0.05, ∗∗*P* < 0.001, Friedman’s test.

**Fig. 3:**
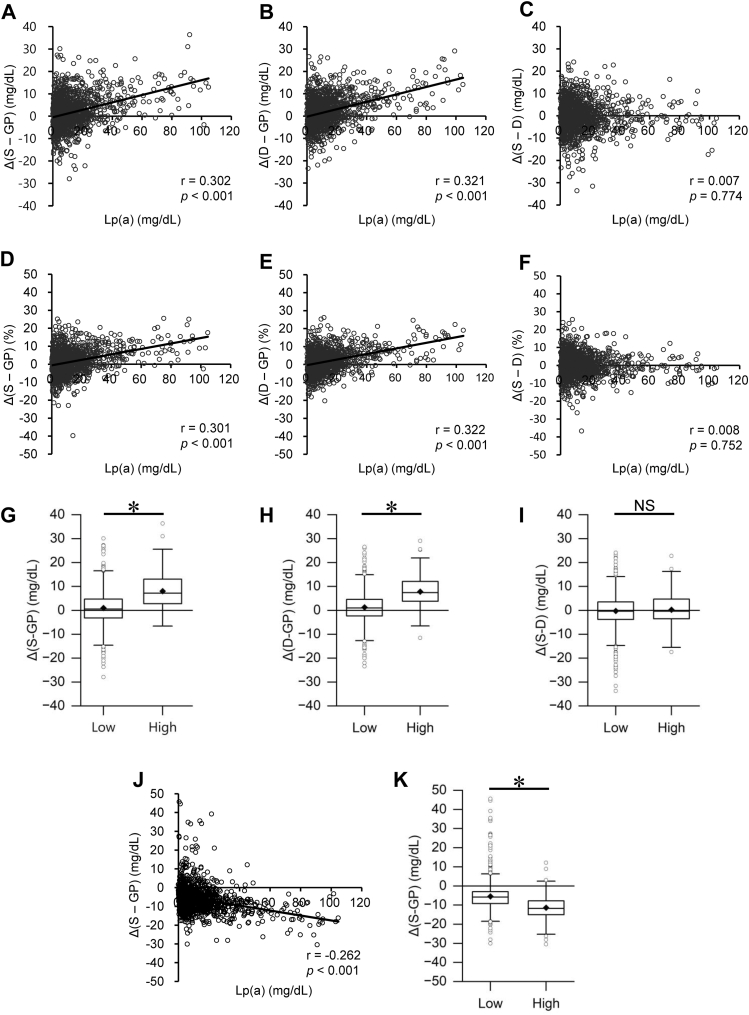
Difference between BQ-based LDL-C and GP-HPLC LDL-C on Lp(a) levels. The association between the LDL-C differences and Lp(a) concentrations was analyzed. The Lp(a) concentration can be tentatively converted to SI units using the formula 3.77 × [x mg/dl] - 2.39 (18). Correlations were evaluated using Spearman’s rank correlation test (A–F). Comparisons of the difference in LDL-C between the low and high Lp(a) groups were assessed. ∗*P* < 0.001, Mann-Whitney *U* test; NS, not significant (G–I). The same analysis was done on VLDL-C (J and K).

**Table 4:** Baseline characteristic of subjects in retrospective data analysis

	Total	Lp(a)<30	Lp(a)>30	*P* value
	N = 1,560	N = 1,421	N = 139	
Age, years	41 (30, 50)	41 (30, 50)	43 (32, 54)	0.050^b^
Male	571 (36.6%)	530 (37.3%)	41 (29.5%)	0.068
Fasting	385 (24.7%)	358 (25.2%)	27 (19.4%)	0.132
Lp(a), mg/dl	7 (4, 16)	6 (3, 13)	45 (36, 61)	<0.001^b^
TC, mg/dl	194 (174, 219)	193 (173, 218)	204 (187, 231)	<0.001^b^
TG, mg/dl	75 (50, 118)	75 (50, 119)	68 (50, 102)	0.199
D-HDL-C, mg/dl	68 (58, 79)	68 (57, 79)	70 (62, 82)	0.017^b^
GP-HDL-C, mg/dl	66 (56, 76)	65 (55, 76)	68 (59, 78)	0.036^b^
D-LDL-C, mg/dl	109 (90, 131)	108 (89, 130)	117 (99, 141)	<0.001^b^
S-LDL-C, mg/dl	109 (89, 131)	108 (89, 130)	119 (97, 140)	<0.001^b^
F-LDL-C, mg/dl^a^	107 (88, 128)	106 (87, 127)	118 (95, 138)	<0.001^b^
GP-LDL-C, mg/dl	107 (88, 129)	107 (88, 128)	111 (93, 131)	0.077
S-VLDL-C, mg/dl	13 (8, 21)	13 (8, 21)	12 (8, 19)	0.509
GP-VLDL-C, mg/dl	20 (15, 29)	20 (14, 28)	24 (20, 32)	<0.001^b^
Body mass index, kg/m^2^	21.7 (19.9, 24.1)	21.7 (19.9, 24.2)	21.2 (19.3, 23.7)	0.036^b^
Waist circumference, cm	77 (70, 84)	77 (70, 84)	75 (69, 82)	0.012^b^
Systolic blood pressure, mmHg	117 (105, 132)	117 (105, 133)	114 (104, 128)	0.149
Diastolic blood pressure, mmHg	72 (63, 82)	72 (63, 82)	72 (63, 80)	0.432
History of smoking	420 (26.9%)	388 (27.3%)	32 (23.0%)	0.277
Under treatment				
Hypertension	99 (6.3%)	92 (6.5%)	7 (5.0%)	0.507
Hyperlipidemia	55 (3.5%)	50 (3.5%)	5 (3.6%)	0.663
Diabetes	30 (1.9%)	27 (1.9%)	3 (2.2%)	0.745
Ischemic heart disease	4 (0.3%)	4 (0.3%)	0 (0.0%)	1.000
Cerebral infarction	4 (0.3%)	4 (0.3%)	0 (0.0%)	1.000
White blood cell count, ×10^3^/μl	5.8 (5.0, 6.8)	5.8 (5.1, 6.8)	5.7 (5.0, 7.1)	0.723
Red blood cell count, ×10^4^/μl	447 (421, 479)	447 (420, 480)	445 (426, 470)	0.417
Hemoglobin, g/dl	13.4 (12.6, 14.7)	13.5 (12.6, 14.7)	13.3 (12.6, 14.1)	0.194
Hematocrit, %	42.2 (39.6, 45.4)	42.2 (39.6, 45.6)	41.7 (39.9, 44.7)	0.452
Platelet count, ×10^4^/μl	23.9 (20.8, 27.0)	23.9 (20.8, 27.0)	23.7 (20.9, 27.2)	0.864
AST, U/L	19 (16, 23)	19 (16, 23)	19 (16, 23)	0.525
ALT, U/L	16 (11, 22)	16 (11, 22)	15 (12, 21)	0.597
ALP, U/L	189 (155, 234)	189 (155, 234)	196 (158, 238)	0.407
γ-GTP, U/L	20 (15, 33)	20 (15, 33)	20 (14, 34)	0.586
Uric acid, mg/dl	4.6 (3.8, 5.7)	4.6 (3.9, 5.7)	4.4 (3.7, 5.4)	0.070
HbA1c, %	5.2 (5.0, 5.4)	5.2 (5.0, 5.4)	5.2 (4.9, 5.5)	0.913

AST, aspartate aminotransferase; ALT, alanine aminotransferase; ALP, Alkaline phosphatase; γ-GTP, gamma-glutamyltransferase; HbA1c, glycated hemoglobin.

a F-LDL-C was analyzed in 1,540 cases (Lp(a) < 30, n = 1,402; Lp(a) > 30, n = 138), excluding participants with elevated TG (≥400 mg/dl).Values are median (quartile 1, quartile 3) or n (%).

b Significant difference.

### Ethics

The study protocol was approved by the institutional research ethics committee of the faculty of medicine of Tokyo Medical and Dental University (currently Institute of Science Tokyo) (approved no. M2015-546 for experimental study, M2022-087 for bioinformatic analysis), The present study was conducted in accordance with the Declaration of Helsinki of the World Medical Association.

## Results

### Simulation of changing density and size of lipoprotein particles by apo(a) binding

Apo(a) molecule is composed of repeated kringle domains (19), so that its molecular weight varies between 300 and 900 kDa. This large protein should significantly influence the density of VLDL. According to the simulated lipoprotein structure proposed by Shen *et al.* (12), the change in the density by attachment of apo(a) was calculated for subfractions of VLDL. As shown in Table 1, apo(a) increases the density of VLDL1 above 1.006 g/ml. Thus, it is conceivable that heavy subfraction of VLDL can be recovered in the bottom fraction of ultracentrifugal separation in the BQ assay if apo(a) is attached. On the other hand, the diameter of the smallest VLDL (VLDL1 in Table 1) is still larger than that of Lp(a) even with the largest apo(a), as shown in Table 2.

**Table 1:** Simulation of change in VLDL density by apo(a) attachment

Apo(a), kDa	VLDL (g/ml)				
	VLDL1	VLDL2	VLDL3	VLDL4	VLDL5
0	0.9917	0.9689	0.9598	0.9616	0.9440
300	1.0013	0.9747	0.9645	0.9652	0.9466
400	1.0043	0.9766	0.9660	0.9664	0.9475
500	1.0075^a^	0.9786	0.9676	0.9676	0.9483
600	1.0103^a^	0.9804	0.9691	0.9687	0.9492
700	1.0131^a^	0.9821	0.9705	0.9698	0.9500
800	1.0161^a^	0.9840	0.9720	0.9710	0.9508

Classification of VLDL particles and their lipid composition are according to simulation by Shen *et al.* (1). Density of each particle (g/ml) was calculated, assuming average density of amino acid 1.34, phospholipid, cholesterol and cholesteryl ester 1.0 and triglyceride 0.9 g/ml.

a Density that might be recovered in the bottom fraction after ultracentrifugation.

**Table 2:** Simulation of relative lipoprotein diameters

Lipoprotein Subclass	Relative Diameter	
	Without apo(a)	With apo(a)
LDL	133.9	149.4 (Lp(a))
VLDL1	198.9	206.5
VLDL2	238.7	243.4
VLDL3	258.5	262.6
VLDL4	282.5	286.3
VLDL5	318.4	321.4

Relative diameter [(volume)ˆ(1/3)] of VLDL, LDL, and Lp(a) (with apo(a) of 900 kDa.), according to lipoprotein structure simulation by Shen *et al.* (1).

### Distribution of apo(a) among lipoproteins defined by density and size

Fresh human serum samples were obtained from three healthy subjects (subject A, B, C). Their basic characteristics and lipid/lipoprotein profiles are summarized in supplemental Table S2 and Table 3, respectively. Subjects A and B had high serum Lp(a) levels of 45 and 83 mg/dl, respectively (167 and 311 nmol/L, converted to SI units, respectively), while Subject C had a lower serum Lp(a) level of 5 mg/dl (16 nmol/L, converted to SI units) (18). It should be noted that there is an interesting finding in LDL-C data among the methods (homogenous assay, BQ method, GP-HPLC, and equation-based methods) with respect to Lp(a) level. For subject A, which has Lp(a) 45 mg/dl, the BQ-based LDL-C values (D-LDL-C, S-LDL-C, F-LDL-C, and BQ-LDL-C) ranged from 105 to 121 mg/dl, whereas the particle size analysis-based LDL-C (GP-LDL-C) was 98 mg/dl. Similarly, for subject B with Lp(a) 83 mg/dl, a difference of LDL-C value was more than 18 mg/dl between the BQ-based LDL-C (122–137 mg/dl) and GP-LDL-C (104 mg/dl). Regarding the apo(a) molecular weight of each subject, we analyzed them using the movement distance on the western blotting profile. As a result, the apo(a) molecular weight was estimated to be 426.6 kDa and 402.1 kDa for Subject A (if two isoforms are present) and 396.4 kDa for Subject B (consistent with a single low molecular weight isoform). These estimated sizes fall within the low to medium apo(a) isoform range, which is strongly associated with elevated plasma Lp(a) concentrations. On the other hand, in subject C, who had a low level of Lp(a) 5 mg/dl, the BQ-based LDL-C ranged from 80 to 89 mg/dl, while GP-LDL-C was 85 mg/dl, showing good matching. Additional apo(a) isoform size analysis was performed in these three subjects (supplemental Table S3). Because this analysis utilized frozen-thawed samples after international shipment, we first verified sample integrity by comparing the Lp(a) levels. Although slight differences in mass concentrations (mg/dl) were observed between the different commercial kits (Sekisui vs. Roche), the calculated molar concentrations after shipment were consistent. Subsequent analysis revealed the exact numbers of KIV repeats and the relative expression ratios of each apo(a) isoform. Although the sample size was limited, subjects with higher Lp(a) levels (Subjects A and B) exhibited smaller predominant apo(a) isoforms (15 and 13 KIV repeats, accounting for 85% and 84% of expression, respectively), whereas the subject with a low Lp(a) level (Subject C) possessed larger isoforms (30 and 34 KIV repeats, supplemental Table S3).

**Table 3:** Lipid and lipoprotein compositions of the human serum used

(mg/dl)	Subject a	Subject B	Subject C
Lp(a)	45 (167 nmol/L)	83 (311 nmol/L)	5 (16 nmol/L)
TC	224	217	156
TG	59	97	39
apoB	77	92	57
D-HDL-C	94	64	60
GP-HDL-C	95	66	61
D-LDL-C	108	122	85
S-LDL-C	120	136	87
F-LDL-C	118.	134	88
BQ-LDL-C	105	137	80
GP-LDL-C	98	104	85
BQ-VLDL-C	4	13	3
S-VLDL-C	10	17	6
GP-VLDL-C	25	52	13

LDL-C and VLDL-C values are determined by direct homogeneous (D), Sampson’s formula (S), Friedewald’s formula (F), modified BQ method (BQ), and gel permeation HPLC (GP).

Distribution of apo(a) in lipoprotein fractions in the BQ method was examined (Fig. 1A-C). The samples were fractionated according to the BQ method by ultracentrifugation at d = 1.006 g/ml, and then the bottom fraction was applied to the sedimentation of apoB. Each fraction was analyzed by electrophoresis-immunoblotting for apo(a) and apoB using the anti-apo(a) monoclonal antibody and anti-apoB antibody. Apo(a) was not detected in the top fraction (d < 1.006 g/ml) representing the VLDL fraction, and all apo(a) was recovered in the bottom fraction (d > 1.006 g/ml) containing LDL and HDL. On the other hand, apoB was detected in both top and bottom fractions. In addition, neither apo(a) nor apoB was detected in the apoB-depleted fraction but were detected in the precipitate in the same pattern as before the precipitation treatment. A similar result was observed for subjects A and B (Fig. 1A, B), whereas for subject C, who had a very low concentration of Lp(a), no apo(a) band was visualized in any fraction (Fig. 1C).

Distribution of apo(a) on lipoproteins based on their particle size, the serum samples were fractionated by GP-HPLC using the LipoSEARCH® system (Fig. 1D–F). The reference position of each lipoprotein was indicated by the cholesterol monitoring peak. Pre-elevated portion of the LDL peak is considered as VLDL, as the fractions 3–7 are VLDL and fractions 8–13 are LDL (5, 10). Each fraction was analyzed by electrophoresis-immunoblotting for apo(a) and apoB. Apo(a) was distributed over the positions of VLDL (fraction 5–7) and LDL, as well as apoB (Fig. 1D, E). These findings, specifically the comparative LDL-C and VLDL-C values by BQ and GP-HPLC for representative subjects, are further exemplified in Table 3. The peak of apo(a) seems a little larger than the apoB peak, which is more parallel with the cholesterol distribution. The finding is consistent that some VLDL contain apo(a)-apoB and apo(a)-bound LDL are slightly larger in Stokes diameter.

To further characterize the isolated lipoproteins, we analyzed the distribution of apoE (a marker for VLDL and its remnants) and apoA-I (a marker for HDL) using western blotting (supplemental Fig. S1). In the ultracentrifugation analysis, the top fraction (d < 1.006 g/ml) showed distinct apoE bands but no apoA-I signal, confirming its identity as the VLDL fraction. The bottom fraction (d > 1.006 g/ml) contained both apoE and apoA-I. Notably, when analyzing the apoB-containing precipitates from the bottom fraction, apoE was clearly detected in subjects A and B (high Lp(a) levels), whereas it was barely detectable in subject C (low Lp(a) level). Regarding the size-based separation by GP-HPLC, apoE was observed in the VLDL fractions (No. 5–7) in Subject B, co-localizing with the apo(a) signal. While apoE was also distributed across the LDL size fractions, the difference in apoE recovery in the density-defined LDL fraction between subjects with high and low Lp(a) was consistent with the findings from ultracentrifugation. Furthermore, apoA-I was exclusively detected in the HDL size fractions, demonstrating an absence of significant contamination of large HDL particles in the LDL fractions.

### Correlations of BQ-based lipoprotein to GP-HPLC lipoproteins

To investigate the impact of Lp(a) on LDL-C measurement discrepancies across a larger population, we analyzed data from a retrospective cohort. The clinical and biochemical characteristics of the study population (n = 1,560) are summarized in Table 4. Notably, the Lp(a) concentrations showed a wide distribution (median, 7 mg/dl; interquartile range [IQR], 4–16 mg/dl), allowing for a robust assessment of its impact on measured LDL-C. The distribution of Lp(a) levels and their relationship with other lipid parameters provided the basis for our subsequent correlation analyses. Correlation was then examined for the BQ-based values of lipoprotein cholesterol to those of GP-HPLCs, that is the density-based values to the size-based values. As for F-LDL-C, we analyzed subjects with TG levels of less than 400 mg/dl (n = 1,540). S-VLDL-C significantly correlated to GP-VLDL-C (r = 0.871, *P* < 0.001) (Fig. 2A). S-LDL-C, D-LDL-C and F-LDL-C highly correlated to GP-LDL-C (r = 0.973, 0.978 and 0.968, respectively, *P* < 0.001) (Fig. 2B–D). All of these BQ-based LDL-C tended to overestimate in comparison to GP-LDL-C (the slopes are higher than 1). Likewise, the values of S-LDL-C and D-LDL-C are both significantly higher than GP-LDL-C using Friedman’s test (*P* < 0.001) (Fig. 2E). Correlation between GP-HDL-C and D-HDL-C was very strong (r = 0.979, *P* < 0.001) (Fig. 2F).

### Influence of Lp(a) on overestimation of LDL-C in the BQ-based assay

The data shown above strongly indicate overestimation of LDL-C based on the BQ assay system, caused by recovery of apo(a)-VLDL in LDL fraction of the ultracentrifugation. On the other hand, apo(a) attached LDL [Lp(a)] seems not recovered in the VLDL fraction in the GP-HPLC method. To confirm this view, we investigated the influence of Lp(a) on the difference between the BQ-based measured LDL-C and GP-LDL-C, for 1,629 human sera collected from the TMDU employees in the regular health check. The difference of BQ-based LDL-C from the GP-LDL-C was analyzed in relation to Lp(a) data (Fig. 3A–C). Likewise, the ratio of change [(BQ-Based LDL-C) - (GP-HPLC LDL-C)]/[GP-HPLC LDL-C] was plotted against Lp(a) (Fig. 3D–F). As mentioned in the method section, the value of this Lp(a) assay is to be considered as molar concentration in the arbitrary unit despite being expressed as mg/dl. As shown in Figure 3A, B, the difference of S-LDL-C and D-LDLC from GP-LDL-C (Δ(S-GP) and Δ(D-GP), respectively) are increased as Lp(a) increased. The difference between S-LDL-C and D-LDL-C (Δ(S-D)) was constant at around zero as Lp(a) increased (Fig. 3C). In order to find if the increase of the difference is due to the increase of LDL, the ratio of change also between the BQ-LDL-C (S-LDL-C and D-LDL-C) and GP-LDL-C (ΔS-GP% and ΔD-GP%, respectively) and between S-LDL-C and D-LDL-C (ΔS-D%) are plotted against Lp(a), demonstrating the similar profile to those of the difference (Fig. 3D–F). To confirm the finding that the difference increases along with the increase of Lp(a) concentration, the difference was compared between the Lp(a) lower 30 mg/dl group (n = 1,421) and the group of 30 mg/dl (n = 139). As shown in Figure 3G, H, the differences are higher in the high Lp(a) group, while no difference was seen in the case of Δ(S-D) (Fig. 3I). The same analysis was performed for VLDL, for S-VLDL-C and GP-VLDL-C. In contrast to the LDL-C analysis, S-VLDL-C is underestimated as the Lp(a) concentration increased (Fig. 3J, K). In conclusion, the higher the Lp(a) molar concentration is, the more the overestimation in the BQ-based LDL-C is, at the expense of underestimation of VLDL.

## Discussion

Our study fundamentally investigates how varying Lp(a) concentrations impact LDL-C and VLDL-C measurements, a critical aspect often overlooked in the accurate assessment of lipid profiles. Our simulation demonstrated that attachment of apo(a) to apoB increases the density of VLDL making some heavy VLDL over 1.006 g/ml. It would result in their recovery in the bottom fraction of ultracentrifuge at 1.006 g/ml. On the other hand, the size of LDL may slightly increase, but not enough to overlap with VLDL, even when bound to the largest apo(a) molecule. These findings were supported by experimental results from ultracentrifugation and GP-HPLC, as well as cohort analysis of 1,629 human samples.

In experimental analyses, no apo(a) was detected in the top fraction of ultracentrifugation, while apoB was found in both top and bottom fractions. Thus, the VLDL particles containing apo(a) are denser than 1.006 g/ml. We acknowledge that the existence of apo(a) within VLDL particles, specifically those with a density >1.006 g/ml, is a novel and less commonly described observation. Our ultracentrifugation data, showing the absence of apo(a) in the 1.006 g/ml supernatant despite apoB presence, strongly supports the notion that apo(a)-containing lipoproteins that would typically be classified as VLDL based on their apoB content, indeed shift to a denser fraction. On the other hand, in lipoprotein separation using GP-HPLC analysis, apo(a) was detected in both the LDL and VLDL fractions. It was suggested that the particle size of LDL with apo(a) (Lp(a)) was larger than that of LDL. While our system defines fractions 3–7 as VLDL and 8–13 as LDL, we acknowledge that the precise boundary can be complex. However, the consistent presence of apo(a) in the earlier-eluting (larger size) fractions alongside VLDL cholesterol peaks, and its distinct behavior from standard LDL, provides compelling evidence that some apo(a) is associated with particles within the VLDL size range, irrespective of exact density cutoffs.

Our analysis of the protein composition provides further mechanistic insight into these density-shifted particles. Specifically, in the apoB-containing precipitates from the ultracentrifugation bottom fraction (d > 1.006 g/ml), which typically contains only LDL, apoE was detected in a manner dependent on the Lp(a) concentration (supplemental Fig. S1). Since apoE is a characteristic component of VLDL and its remnants, these results provide further evidence that apo(a) binds not only to LDL but also to VLDL particles, subsequently increasing their density. The presence of apoC-III and apoE in Lp(a) has been previously reported using immunoaffinity chromatography (20), which showed that apoE-containing Lp(a) is triglyceride-rich and distributes across the VLDL to IDL size range. Our experimental findings align with this, as we detected apoE in the apoB precipitates of the bottom fraction (d > 1.006 g/ml) in an Lp(a)-dependent manner, further supporting the association of apo(a) with VLDL particles. Although apoE can also be distributed in certain LDL subfractions, as previously reported (21, 22, 23), the marked difference in apoE levels between subjects with high and low Lp(a) in this density-defined LDL fraction strongly suggests the presence of VLDL-origin particles that have shifted to a higher density due to apo(a) association. This finding aligns with the density shift observed in ultracentrifugation. It is important to clarify that our interpretation of apo(a) in the VLDL fraction by GP-HPLC refers to its co-elution with particles defined as VLDL by size in our specific system, which, when combined with the density shift, suggests an altered VLDL composition rather than merely Lp(a) migrating with VLDL due to its large size. It is thus speculated definition of LDL-C by BQ-based determination overestimates LDL-C compared to GP-HPLC measurement. This view was confirmed by massive analysis of 1,629 samples of males and females. Statistical analysis of the excess of the BQ-LDL-C to GP-LDL-C showed positive correlation. This was shown at the cost of underestimation of VLDL-C. So, as overall, BQ-based LDL is overestimated compared to GP-LDL-C. The overall particle size-based LDL-C (GP-HPLC) (107 mg/dl) was significantly lower than the density-based LDL-C (S-LDL-C) (109 mg/dl). While this overall mean difference may appear modest, we wish to emphasize that the magnitude of the bias significantly increases depending on the Lp(a) levels, with a maximum observed difference exceeding 30 mg/dl. This is a critical finding: particularly for patients receiving statin therapy—where Lp(a) levels are generally not reduced—a difference of up to 30 mg/dl in measured LDL-C presents a significant challenge for clinical management and target achievement. This overestimation of LDL-C due to Lp(a) interference has direct and serious implications for clinical decision-making. As statin therapy typically does not impact Lp(a) levels, the measured LDL-C in high Lp(a) patients may remain deceptively high even after successful reduction of Lp(a)-independent LDL-C. This discrepancy can result in the misclassification of patients as statin-refractory, potentially leading to the unnecessary escalation of treatment to costly non-statin agents, such as proprotein convertase subtilisin kexin 9 inhibitors, solely based on an analytically biased LDL-C value. Therefore, accurate assessment of Lp(a)-independent LDL-C is essential for appropriate therapeutic monitoring and cost-effective patient care.

Lp(a) concentration used here is based on calculation ignoring variation of molecular weight of apo(a). It is noteworthy that larger apo(a) isoforms, which often correlate with lower Lp(a) concentrations, might mitigate the extent of this error. Simulation in Table 1 suggests that molecular size of apo(a) should significantly influence the magnitude of change in VLDL density by Lp(a) and consequently affect the LDL-C assay based on BQ validation. Accordingly, apo(a) molecular weight data must be considered in addition to its molar concentration to estimate the precise quantitative influence of Lp(a) on BQ-based LDL-C assay values. In this study, we therefore additionally performed an exploratory apo(a) isoform analysis in selected subjects. Notably, even in subjects dominated by low-molecular-weight apo(a) isoforms (approximately 13–15 KIV repeats), we observed a preferential distribution of apo(a) in density-separated LDL fractions, alongside a clear discrepancy in LDL-C measurements (the gap between the density-based reference method and the GP-HPLC method). While our theoretical simulation indicated that an apo(a) mass of 400–500 kDa would only shift VLDL-density particles toward the upper limit of the IDL range (around 1.0075 g/ml), our experimental observations revealed that these particles effectively migrated into the BQ-isolated LDL fraction. Because BQ-isolated LDL inherently encompasses the IDL density range, this physical shift directly causes an overestimation of LDL-C. Although the number of samples analyzed was limited and formal statistical evaluation was not feasible, these observations support our hypothesis. To further refine the correction of this interference, a parameter reflecting both particle number and size, such as ‘molar concentration × molecular weight’, might be more appropriate than using solely molar concentration. Further studies incorporating larger scale apo(a) isoform analyses and additional determinants of Lp(a) particle characteristics will be important to further validate and extend the present findings.

Methods like GP-HPLC, which offer improved specificity in lipoprotein separation, could be considered as potential alternatives or complementary approaches for more accurate LDL-C assessment, especially in individuals with elevated Lp(a). Although circulating Lp(a) is primarily defined by a covalent disulfide bond, we acknowledge the theoretical possibility of non-covalent association of Lp(a) with triglyceride-rich lipoprotein or VLDL, particularly under postprandial or hypertriglyceridemic conditions (24, 25). However, our experimental validation was conducted using fasting, normolipidemic samples, in which such non-covalent associations are reported to be minimal. Additionally, the apo(a) detected in the VLDL fraction by GP-HPLC was co-localized with apoB, suggesting a relatively stable association rather than simple nonspecific adhesion.

As for the experimental validation, only three subjects were analyzed in this study. This small sample size was necessitated by the requirement for high Lp(a) concentrations to ensure reliable detection and separation of apo(a) by the highly demanding GP-HPLC and ultracentrifugation methods. As our retrospective cohort data (n = 1,629) demonstrates, subjects with sufficiently high Lp(a) levels are relatively rare in the Japanese population. While the detailed analysis of these three highly selective individuals provides critical mechanistic insights, this numerical limitation requires caution when generalizing the experimental results to the broader population. Larger, multi-center studies with varied lipid profiles are warranted in the future to further validate these findings.

Furthermore, our study is subject to two additional methodological limitations concerning Lp(a) measurement. First, we were unable to perform chemical dissociation studies (e.g., using L-proline or ε-aminocaproic acid) to definitively assess the nature of apo(a) association with VLDL due to severe sample limitations. Second, the monoclonal immunoassay used to determine Lp(a) concentration has not been compared against the IFCC-endorsed mass spectrometry-based reference measurement procedure, nor could we confirm if the anti-apo(a) antibody utilized is isoform-sensitive. The lack of this gold-standard comparison and detailed antibody characterization introduces potential assay-related variability, which may partly explain the moderate correlation observed between Lp(a) concentration and the BQ-LDL-C bias (r = 0.3). These limitations necessitate caution when interpreting the absolute quantitative relationship.

The present study revealed that LDL-C values obtained from conventional assay methods are overestimated by high Lp(a) levels. To evaluate accurate LDL-C concentrations, quantitative correction is necessary for the standard measurement of LDL-C using a factor of Lp(a) concentrations. However, since quantification of Lp(a) concentration remains issues of accuracy and standardization, further studies are needed to derive a new standard measurement procedure for LDL-C with correction based on Lp(a) concentration.

## Data availability

The data used to support the findings of the present study are available from the corresponding author (Ryunosuke Ohkawa, Graduate School of Medical and Dental Sciences, Institute of Science Tokyo, ohkawa.alc@tmd.ac.jp) upon request.

## Supplemental data

This article contains supplemental data.

## Conflict of interest

The authors declare that they have no conflicts of interest with the contents of this article.

## Figures and Tables

**Yamazaki_Lp(a)_Supporting Information Figure:**
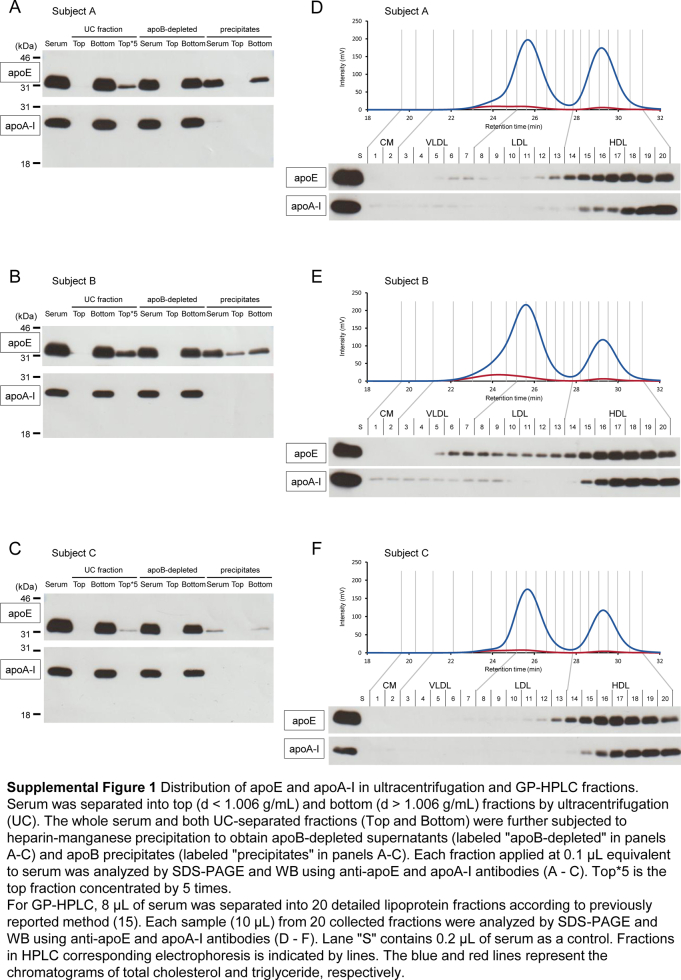


## Supplemental Data

Yamazaki_et_al_JLR_Supporting Information Table
