# Supplementary material for: High lipoprotein(a) results in overestimation of BQ-based low-density lipoprotein-cholesterol measurement
Source: J Lipid Res. 2026 Jun 13;67(7):101080. doi: 10.1016/j.jlr.2026.101080 (PMC13383217; doi:10.1016/j.jlr.2026.101080)
Supplement: Yamazaki_et_al_JLR_Supporting Information Table [file mmc1.docx]

**Supporting Information Tables**

**High lipoprotein(a) results in overestimation of BQ-based low-density lipoprotein-cholesterol measurement**

Azusa Yamazaki^1, 2^, Yuna Hakii^1, 2^, Masumi Ai^3^, Shuji Miyake^4^, Junichiro Takahashi^5^, Akira Yoshimoto^1^, Takahiro Kameda^6^, Naoya Ichimura^2^, Shuji Tohda^2^, Shinji Yokoyama**^7^, Ryunosuke Ohkawa*^1^

1. Clinical Bioanalysis and Molecular Biology, Graduate School of Medical and Dental Sciences, Institute of Science Tokyo, Tokyo, Japan
2. Clinical Laboratory, Institute of Science Tokyo Hospital, Tokyo, Japan
3. Insured Medical Care Management, Graduate School of Medical and Dental Sciences, Institute of Science Tokyo, Tokyo, Japan
4. Health Administration Center of Institute of Science Tokyo, Tokyo, Japan
5. Immuno-Biological Laboratories Co., Ltd, Gunma, Japan
6. Clinical Laboratory Science, Faculty of Medical Technology, Teikyo University, Tokyo, Japan
7. Food and Nutritional Sciences, Chubu University, Kasugai, Japan

Corresponding authors (R Ohkawa*; S Yokoyama**)

*Clinical Bioanalysis and Molecular Biology, Graduate School of Medical and Dental Sciences, Institute of Science Tokyo, 1-5-45 Yushima, Bunkyo-ku, Tokyo 113-8510, Japan. Tel.: +81-3-5803-5375, Fax: +81-3-5803-5375, E-mail: ohkawa.alc@tmd.ac.jp

**Food and Nutritional Sciences, Chubu University, 1200 Matsumoto-cho, Kasugai 487-8501, Japan, Telephone and FAX: +81-569-51-9698, E-mail: syokoyam@fsc.chubu.ac.jp

**Supplemental Table 1. Confirmation of intra-assay repeatability in lipoprotein cholesterol determination by GP-HPLC**

|  | TC (mg/dL) | |
| --- | --- | --- |
|  | Mean ± SD | CV (%) |
| VLDL-C | 28.50 ± 0.41 | 1.44 |
| LDL-C | 101.78 ± 0.71 | 0.71 |
| HDL-C | 83.93 ± 0.35 | 0.42 |

The same serum sample was injected 20 times consecutively to quantify lipoprotein cholesterol (n = 20).

TC, total cholesterol; SD, standard deviation; CV, coefficient of variation.

**Supplemental Table 2. Basic information of three healthy subjects**

|  | Subject A | Subject B | Subject C |
| --- | --- | --- | --- |
| Sex | Female | Male | Female |
| Age, years | 24 | 35 | 30 |
| Body mass index, kg/m^2^ | 19.8 | 18.0 | 21.5 |

**Supplemental Table 3. Additional Lp(a) measurements and apo(a) isoform analysis**

|  | | Subject A | Subject B | Subject C |
| --- | --- | --- | --- | --- |
| Lp(a) particle number (nmol/L) | | 150.6 | 308.8 | 10.3 |
| Lp(a) Mass (mg/dL) | | 69.8 | 146.6 | 6.3 |
| Isoform 1 | (KIV Motifs) | 15 | 13 | 30 |
|  | (% expression) | 85% | 84% | 60% |
| Isoform 2 | (KIV Motifs) | 34 | 27 | 34 |
|  | (% expression) | 15% | 16% | 40% |

Lp(a) was measured using the Roche Lp(a) Gen2 kit on a Roche c502 analyzer. Molar concentrations were obtained using a calibrator set traceable to WHO PRM 2B, and mass concentrations were determined using the Roche internal standards. KIV, kringle IV.
